# City-level building operation and end-use carbon emissions dataset from China for 2015–2020

**DOI:** 10.1038/s41597-024-02971-4

**Published:** 2024-01-26

**Authors:** Yanhui Yu, Kairui You, Weiguang Cai, Wei Feng, Rui Li, Qiqi Liu, Liu Chen, Yuan Liu

**Affiliations:** 1 School of Management Science and Real Estate, Chongqing University, Chongqing, 400044 China; 2 Institute for Carbon Neutrality Technology, Chinese Academy of Sciences – Shenzhen Institute of Advanced Technology, Shenzhen, 518055 China

**Keywords:** Energy and society, Energy management, Environmental impact

## Abstract

The building sector, which accounts for over 20% of China’s total energy-related carbon emissions, has great potential to reduce emissions and is critical to achieving China’s emissions peak and carbon neutrality targets. However, the lack of data on operational carbon emissions and end-use carbon emissions in the building sector at the city level has become a major barrier to the development of building energy conservation policies and carbon peaking action plans. This study uses a combination of “top-down” and “bottom-up” methods to account for the operational carbon emissions of buildings in 321 cities in China from 2015 to 2020. The energy consumption in buildings is further broken down into six end uses: central heating, distributed heating, cooking and water heating (C&W), lighting, cooling, appliances and others (A&O). The dataset can serve as a reference to support city-level policies on peak building emissions and is of great value for the improvement of the carbon emissions statistical accounting system.

## Background & Summary

The building sector is one of the three largest sectors of energy consumption and carbon emissions^1^. In 2021, worldwide building activity returned to prepandemic levels, and carbon emissions from building operations reached an all-time high of approximately 10 billion tonnes, accounting for 27% of total global energy-related emissions^2^. In 2020, China’s building operations activities generated 2.16 billion tonnes of emissions, accounting for 22% of the country’s total emissions^3^, and this share is likely to continue to rise in the future^4–6^. The Chinese government set a “30–60” emissions target for carbon peaking and carbon neutrality in 2020, and the Ministry of Housing and Urban‒Rural Development (MOHURD) issued the *Implementation Plan for Peak Carbon in Urban and Rural Construction* in 2022. With the continuous improvement of the national and provincial plans for carbon peak implementation for the building sector^7,8^, the work to achieve a carbon peak in buildings will gradually be implemented at the city level, but the serious lack of data on carbon emissions in buildings at the city level is a constraint to the implementation of this work.

As cities are the main focus of human energy consumption and emissions^9^, generating more than 70% of global emissions and serving as the main implementers of various climate mitigation policies^10^, the development of emission inventories has in recent years focused more on the city level^11^. The compilation of China’s emission inventories is becoming increasingly perfected, and some organizations have already compiled more comprehensive city-level accounts of China’s carbon emissions through extensive data collection, such as Carbon Emission Accounts and Datasets (CEADs), which compiled an emissions inventory of 297 cities for the period 1997-2020^10,12,13^, and the China City Greenhouse Gas Working Group (CCG, http://www.cityghg.com/toCauses?id=4), which compiled an emission inventory of 340 cities across China^14,15^. Others have accounted for city energy consumption and carbon emissions using spatial high resolution^16^, night-time lighting simulations^17,18^, input‒output analysis^19^ and so on.

Existing studies have used both “top-down” and “bottom-up” approaches to account for carbon emissions from buildings at the national and provincial levels in China^20^ but have not yet gone as far as the full city level. Furthermore, as energy consumption and carbon emissions during the operational phase of a building are generated through several specific end-use activities (e.g., heating, cooling, cooking, etc.)^21,22^, it is important to understand the anticipated level of activity and the intended structure of energy use for planning and deploying building energy conservation^23^. While some institutions and scholars have analysed the end-use emissions of Chinese buildings based on both macrodata breakdowns and large-scale household surveys^24–29^, other studies have been conducted only for specific cities or regions^30–32^ and have focused more on the heating and space cooling components of buildings in energy accounting and scenario modelling^33–36^.

In general, existing studies and emission inventories have the following shortcomings, which do not support emission reduction efforts in the building sector at the city level. **(1) It is not possible to calculate the complete building carbon emissions based on existing emission inventories**. Most emission inventories are developed based on a production-side perspective^10,14^. Although these inventories can distinguish between several economic sectors and residential sectors and can calculate direct fossil energy emissions from the building sector, electricity emissions, which account for 50% of total building emissions, and central heating emissions, which account for 20% of total building emissions, are attributed to the production and supply sector of electricity and heat and are not reflected in end consumption. **(2) There is a lack of a comprehensive city-level methodological system for tracking carbon emissions from buildings**. Most of the existing city-level studies on carbon accounting in buildings are based on a few major cities such as Beijing, Shanghai and Guangzhou and are not sufficiently comprehensive^37–39^. At the same time, the commonly used downscaling approach computes building energy consumption as a proportion of the city’s population and economy^40^, making it difficult to reflect the characteristics of the building sector and bringing greater uncertainty in the accounting results^41^. **(3) There is a lack of a clear approach for accounting for building end-use energy consumption and carbon emissions**. The methodologies for splitting end-use energy consumption in buildings have not been explained in detail in previous studies, and the results of the various types of splitting studies suffer from large data differences and undisclosed data, providing limited support for city and regional energy conservation efforts aimed at buildings.

To support the preparation of city-level policies for carbon peaks in the building sector and to promote the improvement of the carbon accounting system, based on national and provincial building carbon accounting methods, this study uses a combination of “top-down” and “bottom-up” methods to account for the energy consumption and carbon emissions of building operations in 321 cities from 2015 to 2020 through an extensive collection of statistical data. The emissions data were further broken down into six end uses: central heating, distributed heating, cooking and water heating (C&W), lighting, cooling, appliances and others (A&O).

## Methods

**Fig. 1:**
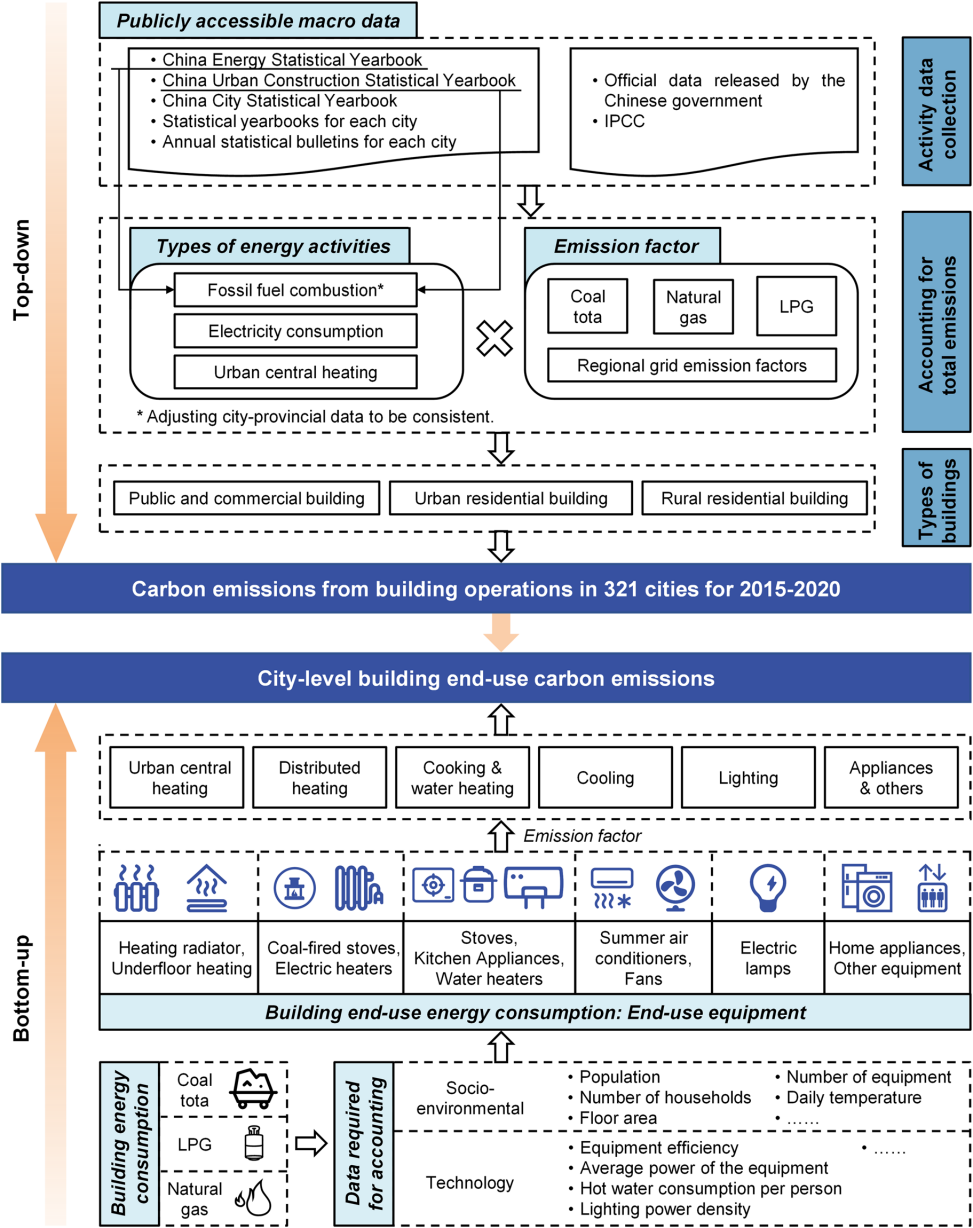
Framework for accounting for building operations and end-use carbon emissions at the city level.

The process of accounting for building operations and end-use carbon emissions at the city level in China is illustrated in Fig. 1. In essence, we acquire city-level energy consumption data from public sources such as statistical yearbooks and official government reports. Subsequently, a method similar to Energy Balance Sheet (EBS) splitting, coupled with energy emission factor data from government and IPCC organizations, is employed to calculate the operational carbon emissions of various building types in a “top-down” approach. Next, utilizing socio-economic data such as population, floor area, temperature, and technical parameters like equipment efficiency, we determine the energy consumption of various equipment such as air conditioners, lights, stoves, and water heaters in a “bottom-up” manner. This information is then aggregated with the emission factor data to generate carbon emission data for six types of building end-uses: central heating, distributed heating, C&W, lighting, cooling, and A&O.

### City boundaries and accounting scopes

In this study, “cities” refers to the four municipalities directly under the central government and the prefecture-level administrative regions in China, which refers to all second-level administrative regions, including prefecture-level cities, prefectures, autonomous prefectures and leagues. To ensure uniformity and comparability of data, the administrative divisions at the prefectural level are unified using the 2020 classification. Our accounting objects cover a total of 321 cities that cover 98.6% (1.392 billion) of China’s population (1.412 billion) and 99.2% (99.71 trillion yuan) of its GDP (100.55 trillion yuan) in 2020^3^.

Carbon emissions from buildings in this study refer to the carbon emissions generated by the energy consumed during the operational phase of buildings to meet the needs of human activities (e.g., heating, cooling, lighting, cooking, office operations, etc.), which can be classified into direct emissions (e.g., emissions from the combustion of coal, oil and gas) and indirect emissions (e.g., emissions from the use of electricity and central heating) depending on the nature of the activity. Buildings refer to civil buildings, including urban residential buildings, rural residential buildings and public and commercial buildings (P&C), which can be roughly compared to “Urban residential”, “Rural residential” and “Wholesale and Retail Trades, Hotels and Catering Services + Others”, respectively, in the end consumption sector of China’s EBS^42^. As China’s energy statistics system does not include noncommodity energy consumption, such as solar energy and biomass, and as biomass is often treated as a zero-emission energy source in research, we only consider the consumption of commodity energy in buildings in our energy consumption calculations.

Energy consumption in buildings mainly comes from various types of appliances such as air conditioners, refrigerators, and televisions and various types of equipment such as radiators, light bulbs, and lifts in buildings. In particular, the rural areas of northern China and the hot summer and cold winter regions south of the Qinling Mountains-Huaihe River Line are characterized by extensive use of distributed heating in winter, which is significantly different from the central heating in northern towns^27^. Therefore, this study splits the space heating of buildings into central heating and distributed heating and ultimately identifies six building end-use behaviours: central heating, distributed heating, cooking and water heating (C&W), cooling, lighting, appliances and others (A&O)^43^.

### Carbon emissions from building operations

Coal total (including raw coal, all types of washed coal, and briquettes), liquefied petroleum gas (LPG), and natural gas (NG) are the three main types of fossil energy used in the building operation phase, and their energy consumption accounts for more than 90% of the direct fossil energy consumption in building operation in China^3^. Therefore, this study considers these three types of fossil energy sources to account for direct fossil energy emissions (*CE*_*direct*_) from city building operations. Indirect electricity emissions (*CE*_*elec*_) are accounted for through building electricity consumption and regional grid emission factors. Indirect central heating emissions (*CE*_*c–heating*_) are accounted for through energy consumption at the heat source. The accounting for carbon emissions (*CE*) from city buildings is shown in Eq. 1.

1 $$\begin{array}{l}CE=C{E}_{direct}+C{E}_{elec}+C{E}_{c-heating}=\sum _{t}({E}_{direct,t}\times C{F}_{t})+{E}_{elec}\times C{F}_{elec}+\sum _{t}({E}_{c-heating,t}\times C{F}_{t})\end{array}$$

where *E*_*direct,t*_ is the three types of fossil energy consumed directly by building operations, *CF*_*t*_ is the carbon emission factor of energy, *E*_*elec*_ is the electricity consumed by building operations, *CF*_*elec*_ is the carbon emission factor of the regional grid in the area where the city is located, and *E*_*c–heating,t*_ is the energy consumed by heating plants for central heating, including fossil energy and electricity.

As shown in Table 1, the carbon emission factors for each type of fossil energy are available through a variety of authoritative sources, such as the IPCC and the National Development and Reform Commission (NDRC). For electricity, this study uses the average carbon emission factor of China’s regional power grid, which is updated year by year. The coverage of the power grid is referenced to the Ministry of Ecology and Environment of the People’s Republic of China (MEE)^44^, and the accounting method is based on the EBS, as shown in Eq. 2.

2 $$C{F}_{elec}=\frac{{\sum }_{n}{\sum }_{t}\left({E}_{ele{\prime} ,n,t}\times C{F}_{t}\right)}{{\sum }_{n}\left(EL{E}_{fire,n}+EL{E}_{clean,n}\right)}$$

where *E*_*ele*′*,t*_ is the fossil energy consumed for thermal power generation, *n* is the province included in the regional grid, and *ELE*_*fire,n*_ and *ELE*_*clean,n*_ are the province’s thermal power generation and clean energy primary generation, respectively.

**Table 1:** Carbon emission factors for energy and regional grids in China.

Types of energy		Emission factor						Source
Coal total		2.66 tCO_2_/tce						^55^
Liquefied petroleum gas (LPG)		3.11 tCO_2_/t						^57,64^
Natural gas (NG)		2.16 tCO_2_/m^3^						^57,64^
Regional power grid (kgCO_2_/kWh)	**Region**	**2015**	**2016**	**2017**	**2018**	**2019**	**2020**	^3,65^
	North	0.844	0.821	0.820	0.795	0.783	0.761	
	Northeast	0.909	0.856	0.851	0.790	0.803	0.732	
	East	0.648	0.625	0.629	0.618	0.596	0.579	
	Central	0.443	0.418	0.410	0.421	0.416	0.380	
	Northwest	0.663	0.656	0.651	0.624	0.612	0.612	
	South	0.414	0.409	0.426	0.420	0.409	0.406	

After that, the key issue in accounting is to determine the energy consumption of each category of city buildings.

#### Fossil energy

The *China Urban Construction Statistical Yearbook* (CUCSY) lists the amount of LPG and NG consumed by residential households in all Chinese cities for all years, which can be used as a partial basis for accounting for fossil energy consumption in residential buildings. Assuming that the proportion of energy consumed by the three building types in a city is the same as the province in which it is located, LPG and NG consumption in P&C is determined based on the fossil energy consumption of the different building types in the provincial EBS. In particular, as city coal consumption data are not compiled in any of the various statistical yearbooks and most cities do not publish coal consumption data for different sectors, we refer to previous studies to derive coal consumption for different building types in cities based on building coal consumption data in the provincial EBS using relevant socioeconomic indicator weights^10,40^. This approach is also used to address the issue that the sum of LPG and NG consumption in cities within a province in CUCSY is much smaller than the values given in the provincial EBS. Despite the uncertainties in this approach, this method is still the most accurate at this stage^14^.

3 $${E}_{direct,t}={E}_{direct,t}^{province}\times {P}_{t}$$

4 $${P}_{t}=\frac{Inde{x}_{city}}{Inde{x}_{province}}\times 100{\rm{ \% }}$$

where ${E}_{direct,t}^{province}$ is the fossil energy consumption of buildings in the provincial EBS, *P*_*t*_ is the city-province ratio. When *t* = *LPG or NG*, *Index*_*city*_ is the consumption of energy *t* by buildings in the city obtained from CUCSY; *Index*_*province*_ = Σ*Index*_*city*_ is the sum of all cities in the province where the city is located. When *t* = *Coal tatal*, *Index*_*city*_ and *Index*_*province*_ represent the socioeconomic indicators of the city and its province, respectively, with the indicator for residential buildings being population and the indicator for P&C being the value added of the tertiary sector.

#### Electricity

Electricity carbon emissions account for more than 50% of the total carbon emissions from building operations in China and are the most important factor affecting carbon emissions^3^. Referring to the EBS split for estimating building energy consumption in previous studies^42^, the following formula can be used to calculate electricity consumption in city buildings:

5 $${E}_{elec}={E}_{elec,UR}+{E}_{elec,RR}+{E}_{elec,WRHCO}+\alpha \times {E}_{elec,TSP}$$

where *UR* and *RR* are urban residential and rural residential, respectively, *WRHCO* is wholesale and retail trades, hotels and catering services, and others, *TSP* is transport, storage and post, and *α* is the proportion of electricity used for construction in *TSP*, which is generally taken as 40% in China.

We manually collected and aggregated electricity consumption data by sector for most cities through publicly available data (e.g., statistical bulletins, statistical yearbooks, statistical news releases from city governments and statistical bureaus, etc.) and by writing inquiries, as shown in Table 2. Missing data were dealt with in 2 ways: (1) Interpolation: for cities with missing data for intermediate years, linear interpolation was used to complete the data. For example, Zhengzhou’s statistical bulletin published electricity consumption for residential use in 2016 and 2018, but not 2017 data, and sought the average value of the two years 2016 and 2018 as the electricity consumption data for 2017. (2) Proportional calculation: Since the *China City Statistical Yearbook* provided the total social electricity consumption data for almost all cities, the assumption that a city has the same energy consumption intensity as the province it is located in is no longer applicable. For cities with completely missing sectoral electricity consumption data, it was assumed that they share the same electricity consumption structure as the province they are located in. These proportions of electricity consumption included electricity consumption in the transport sectors as a ratio of electricity consumption in the tertiary industries, electricity consumption in residential life as a ratio of electricity consumption in society as a whole, and electricity consumption in urban residential life as a ratio of electricity consumption in residential life. In this way, the electricity consumption in buildings was divided from the amounts listed in total statistics.

**Table 2:** Number and percentage of cities with available data on electricity consumption in different sectors.

Year	Urban Residential		Rural Residential		Tertiary sector		Transport, Storage and Post	
	Number of cities	Share	Number of cities	Share	Number of cities	Share	Number of cities	Share
2015	151	45%	152	46%	162	49%	112	34%
2016	158	47%	159	48%	182	55%	113	34%
2017	301	90%	220	66%	185	56%	112	34%
2018	298	89%	217	65%	184	55%	111	33%
2019	298	89%	219	66%	192	58%	118	35%
2020	174	52%	172	52%	187	56%	111	33%

#### Central heating

Central heating is the main method of heating in northern urban areas in winter and refers to supplying production and domestic heat from one or more heat sources to heat users in the city through a heat network, which requires a certain scale. The energy consumption of central heating in buildings (*E*_*c–heating*_) is the sum of the energy consumption of boilers (*E*_*boiler*_), cogeneration (*E*_*cogeneration,caol*_) and heat pumps (*E*_*other,elec*_), which can be accounted for by using the heat supply and energy intensity of different central heating technologies.

6 $${E}_{c-heating}=\sum _{t}{E}_{boiler,t}+{E}_{cogeneration,coal}+{E}_{other,elec}$$

7 $${E}_{boiler}={E}_{boiler,NG}+\left({H}_{boiler}-{E}_{boiler,NG}\times {\eta }_{boiler,NG}\right)\times HC{C}_{boiler,coal}$$

8 $${E}_{cogeneration,coal}={H}_{cogeneration}\times HC{C}_{cogeneration,coal}$$

9 $${E}_{other,elec}=\left[{H}_{total}-\left({H}_{cogeneration}+{H}_{boiler}\right)\right]\times HE{C}_{other}$$

where *H*_*total*_, *H*_*boiler*_ and *H*_*cogeneration*_ are the total central heating capacity of the city, heat supply from boilers and heat supply from cogeneration plants, respectively. *E*_*heating,NG*_ is the energy consumption of NG used for central heating, *η*_*boiler,NG*_ is the thermal efficiency of gas boilers, here calculated at 95%, *HCC*_*boiler,coal*_ is the heating coal consumption of coal-fired boilers, calculated at 80% thermal efficiency, which is 42.7 kgce/GJ. *HCC*_*cogeneration,coal*_ is the combined heating coal consumption of cogeneration, referring to the results of the exergy sharing method^45^ and considering that the heating method is closer to that of coal-fired boilers in winter when cogeneration plants face higher heating demand; it is also assumed that the heating coal consumption of cogeneration plants in winter is the average value of both, 31.7 kgce/GJ. *HEC*_*other*_ is the comprehensive heating energy consumption of various types of heat pumps for central heating, which is taken as 25.9 kgce/GJ^45^.

It should be noted that the data on central heating areas in cities provided in CUCSY are underreported to varying degrees. Beijing, which has a better statistical system, undercounted more than 30% of its centralized heat supply area in 2020^46^, which would lead to an underestimation of the corresponding heat supply. Therefore, in this study, the central heating volume is uniformly expanded by 30%.

### End-use energy and carbon emissions for building operations

Of the five remaining end-use behaviours other than central heating, three—cooling, lighting and A&O—consume only electricity. Distributed heating requires the use of nonelectric heating equipment such as coal stoves and boilers and electric equipment such as electric heaters and electric blankets, consuming coal and electricity. C&W requires the use of gas stoves, cellular coal stoves and electric cooking appliances (such as rice cookers), consuming coal, NG, LPG and electricity. Noncommodity energy sources such as solar and biomass are not accounted for in this study.

#### Cooling

The limit values of energy consumption indicators for summer air conditioning, calculated by the relevant building energy design standards, can be much higher than the actual survey values^47^, so this study devised a macroscopic approach to calculating building cooling electricity consumption. First, the average temperature of each province was calculated in half-month intervals, and then based on national standards^48^, defining the time below 10 °C as winter, the time above 22 °C as summer, and the time in between as spring and autumn. Using month-by-month electricity consumption data from the building sector, after determining the duration of the four seasons in the different provinces, the average monthly electricity consumption in summer, minus the average monthly consumption in spring and autumn, was multiplied by the summer duration, and the result was used as representative of summer cooling electricity consumption for the different building types in the province. Finally, the proportion of provincial electricity consumption for cooling for different building types to total electricity consumption ($P{E}_{elec,j,m}^{cooling}$) was calculated and applied to the city level to calculate cooling electricity consumption (${E}_{elec}^{cooling}$).

10 $$P{E}_{elec,j,i,m}^{cooling}=P{E}_{elec,j,m}^{cooling}=\left(M{E}_{j,m}^{summer}-M{E}_{j,m}^{spring\,\& \,autumn}\right)\times {D}_{j,summer}/{E}_{elec,j,m}$$

11 $${E}_{elec}^{cooling}=\sum _{m}{E}_{elec,j,i,m}^{cooling}=\sum _{m}\left({E}_{elec,i,m}\times P{E}_{elec,j,i,m}^{cooling}\right)$$

where *i* and *j* are a city and its province, respectively, *m* is the three types of buildings, *ME* is the average monthly electricity consumption of the season, *D* is the duration of the season with a minimum unit of half a month, and *E*_*elec,i,m*_ is the electricity consumption of building *m* in city *i*.

#### Lighting

With reference to the common calculation method for the energy consumption of building lighting systems, the annual electricity consumption of city building lighting (${E}_{elec}^{lighting}$) is calculated using the lighting power density, monthly lighting hours and building lighting area for different buildings.

12 $${E}_{elec}^{lighting}=\sum _{m{\prime} }{E}_{elec,i,m{\prime} }^{lighting}=12\sum _{m{\prime} }\left({P}_{m{\prime} }\times {t}_{m{\prime} }\times L{A}_{i,m{\prime} }\right)$$

where *m*’ refers to 9 types of buildings (residential, office, retail, hotel, restaurant, school, hospital, entertainment, and others), which all belong to P&C, except for residential buildings. ${P}_{m{\prime} }$ is the building lighting power density; ${t}_{m{\prime} }$ is the building monthly lighting hours; and $L{A}_{i,m{\prime} }$ is the building lighting area.

#### Cooking and water heating (C&W)

In the assumptions of this study, NG and LPG are only used for C&W. In addition, some electricity and coal consumption is for this end use. Electricity consumption for water heating is calculated based on the commodity energy demand for water heating in city buildings, the share of electric water heaters and the efficiency of electric water heaters. Electricity consumption for cooking is calculated based on the number of households and the average household electricity consumption of electric cookers.

13 $${E}^{{\rm{C}}\& {\rm{W}}}={E}_{LPG}+{E}_{NG}+{E}_{elec}^{{\rm{C}}\& {\rm{W}}}+{E}_{coal}^{{\rm{C}}\& {\rm{W}}}$$

14 $${E}_{elec}^{{\rm{C}}\& {\rm{W}}}={E}_{elec}^{water}+{E}_{elec}^{cooking}=E{D}^{water}\times {P}_{elec}^{{\prime} }/{\eta }_{elec}+H\times {e}_{cooking}$$

15 $$E{D}^{water}=HWC\times {\rho }_{water}{c}_{water}\left({t}_{h}-{t}_{1}\right)\left(1-{P}_{solar}\right)$$

where *E*^*C*&*W*^ is the energy consumption for C&W in city buildings, *E*_*LPG*_ and *E*_*NG*_ are building LPG and NG consumption, respectively, ${E}_{elec}^{C\& W}$ and ${E}_{coal}^{C\& W}$ are electricity and coal used for C&W, respectively, and *ED*^*water*^ is the commercial energy demand for building water heating. $P{{\prime} }_{elec}$ is the proportion of household electric water heaters to nonsolar water heaters, and *η*_*elec*_ is the thermal efficiency of electric water heaters, taken as 95%. *H* is the number of households, and *e*_*cooking*_ is the annual electricity consumption of household electric cookers. *HWC* is the annual hot water use in city buildings, calculated based on the daily hot water quota per resident, public service water consumption and the proportion of hot water use in P&C. *ρ*_*water*_ is the hot water density, and *c*_*water*_ is the specific heat capacity of hot water, taken as 4.187 kJ/(kg·°C). *t*_*h*_ is the designated temperature of hot water, taken as 60 °C, and *t*_1_ is the designated temperature of cold water in the city, taken as the annual average temperature of the city. *P*_*solar*_ is the share of solar water heaters.

**Fig. 2:**
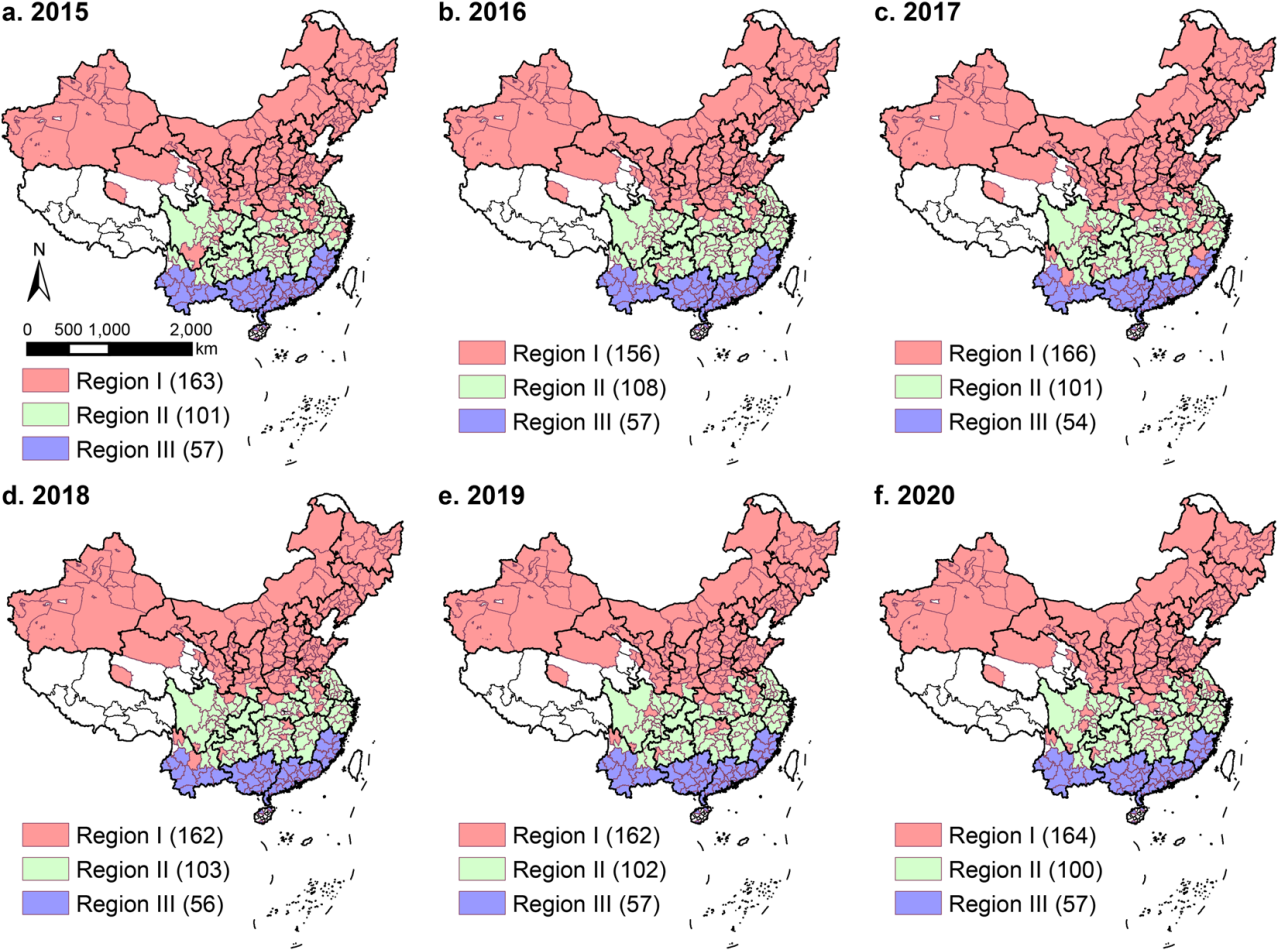
Three types of Chinese regions classified by heating behavior and temperature and the number of cities in each region in 2015–2020. Region I denotes areas with central heating, where heating activities include both central and distributed heating; Region II denotes areas with only distributed heating; and Region III denotes areas with no heating activities and average monthly temperatures above 10 °C. The annual zoning will change due to changes in central heating in cities, which mainly occurs in the provinces of Jiangsu, Anhui, and Sichuan. White areas in the figure indicate cities and regions not included in the study.

When ${\eta }_{gas}\left({E}_{LPG}+{E}_{NG}\right)+{\eta }_{elec}{E}_{elec}^{water} < E{D}^{water}$, coal is used as a supplementary energy source to ensure that the energy demand for water heating is met, that is, to ensure that Eq. 16 holds. In addition, the remaining bulk coal in buildings in cities belonging to Region III is allocated to cooking to obtain full coal consumption for the C&W (${E}_{coal}^{C\& W}$). The building zoning of Chinese cities based on heating activities is shown in Fig. 2, Supplementary Tables S1 and S2.

16 $$E{D}^{water}={\eta }_{gas}\left({E}_{LPG}+{E}_{NG}\right)+{\eta }_{elec}{E}_{elec}^{water}+{\eta }_{coal}{E}_{coal}^{water}$$

where ${E}_{coal}^{water}$ is the coal consumption for water heating in buildings and *η*_*gas*_ and *η*_*coal*_ are the thermal efficiencies of gas-fired water heaters and coal-fired stoves, respectively, taken as 90% and 40%.

#### Distributed heating

Energy consumption for distributed heating (*E*^*d–heating*^) in city buildings comes from heating appliances and bulk coal. The accounting for electricity used for distributed domestic heating is similar to the accounting for electricity used for cooling, in that the difference between winter and “spring and autumn” electricity consumption is used to determine the proportion of electricity used for heating to the total building electricity consumption ($P{E}_{elec,j,m}^{d-heating}$) and thus to determine the energy consumption of appliances for heating (${E}_{elec}^{d-heating}$). The difference between total coal consumption and coal consumption for C&W is considered to be the coal consumption for distributed heating (${E}_{coal}^{d-heating}$).

17 $${E}^{d-heating}={E}_{elec}^{d-heating}+{E}_{coal}^{d-heating}$$

18 $$P{E}_{elec,j,i,m}^{d-heating}=P{E}_{elec,j,m}^{d-heating}=\left(M{E}_{j,m}^{winter}-M{E}_{j,m}^{spring\& autumn}\right)\times {D}_{j,winter}$$

19 $${E}_{elec}^{d-heating}=\sum _{m}{E}_{elec,j,i,m}^{d-heating}=\sum _{m}\left({E}_{elec,i,m}\times P{E}_{elec,j,i,m}^{d-heating}\right)$$

20 $${E}_{coal}^{d-heating}=\sum _{m}{E}_{elec,i,m}^{d-heating}=\sum _{m}\left({E}_{coal,i,m}-{E}_{coal,i,m}^{C\& W}\right)$$

#### Appliances and others (A&O)

The energy use of appliances and facilities in buildings is greatly influenced by occupant behaviour, and even though the *China Statistical Yearbook* provides data on the average household ownership of some major appliances such as TVs, washing machines and refrigerators, data on the power and usage habits of household appliances lack large-scale surveys, so accounting for energy use on an equipment-by-equipment basis can be subject to large uncertainties. As electricity is involved in all building end uses, we subtract the sum of other end uses from the total electricity consumption, which is the *A&O* energy use (${E}_{ele,i}^{A\& O}$).

21 $$\begin{array}{l}{E}_{ele}^{A\& O}=\sum _{m}{E}_{elec,m}^{A\& O}=\sum _{m}({E}_{elec,m}-{E}_{elec,m}^{cooling}-{E}_{elec,m}^{lighting}-{E}_{elec,m}^{C\& W}-{E}_{elec,m}^{d-heating})\end{array}$$

### Data source

All source data entered in this study in the total accounting and end-use breakdown were obtained from official agencies and relevant academic institutions and are publicly available. In carbon accounting, CUCSY provides the following data by city: NG sales for central heating, total heat supply by technology type (cogeneration plants, boilers and heat pumps) and heat supply area by building type (residential buildings and P&C). For end use, data on city population, number of households, and residential building area are obtained from subcounty data from China’s 6th and 7th population censuses, and P&C floor area is extrapolated based on the building stock turnover model^49^. Data on average daily temperatures for Chinese cities and provinces over the years are obtained from the National Oceanic and Atmospheric Administration (NOAA, https://www.ncei.noaa.gov/data/global-summary-of-the-day/archive/), and submonthly electricity consumption data at the provincial level are obtained from provincial statistical bureaus (Supplementary Table S3); lighting power density and monthly lighting hours in buildings are from *Standard for building carbon emission calculation* and *General code for energy efficiency and renewable energy application in buildings*^50,51^; the daily water consumption quota for hot water per person is from *Standard for design of building water supply and drainage*^52^, the water consumption for public services is from CUCSY, and the proportion of hot water consumption in P&C is from *Standard for water saving design in civil building*^53^. Data on the proportion of electric and solar water heaters applied and the average household electricity consumption of electric cookers are from *Chinese Household Energy Consumption Report 2016* and *2017 Annual Report on China Building Energy Efficiency*^26,29^; data on the operational efficiency of various types of water heaters are from *2021 Annual Report on China Building Energy Efficiency*^27^.

## Data Records

The dataset generated in this study is freely accessible and downloadable at the public repository Figshare^54^.

An Excel file containing 12 worksheets by year was compiled to present the dataset. The dataset records carbon emissions from building operations in 321 cities in China from 2015–2020 and contains data on total carbon emissions, carbon emissions from three building types, carbon emissions from three energy activity categories and carbon emissions from six end-use categories. The unit of emission in the dataset is 10,000 tons of CO_2_. In addition, in order to ensure the rigor of the dataset, we marked the cities in which we filled in the missing electricity consumption data in the provided data files, and explained in detail the categories of the filled-in data. The results of the Monte Carlo uncertainty analyses for all years are provided along with the data files.

## Technical Validation

### Data comparison

The method of using EBS splitting to account for national and provincial building operational carbon emissions is well established and widely used by MOHURD, MEEPRC, and some provinces and cities in their carbon accounting work^55^. However, the compilation of city-level EBS in China is poor, with only a very small number of cities publishing EBS, and most of the published data are older than 2015, making it difficult to account for city-level operational building carbon emissions data beyond 2015. After searching, seven cities (excluding municipalities directly under the central government) were identified that published a full EBS between 2015 and 2020. Therefore, to verify the validity of the method of accounting for the data in this study, we applied both accounting methods to these cities and compared the differences in calculation results between the different accounting methods.

As shown in Table 3, the deviation of carbon emission accounting for buildings in all seven cities is within 10%. The carbon emissions in some of the cities we account for are lower than the amount accounted for using the EBS, mainly because we ignore emissions from diesel consumed in P&C. At the national level, the carbon emissions from diesel in the operational phase of buildings in 2020 will be approximately 28.83 million tonnes, representing only 1.3% of total carbon emissions from buildings^3^, and ignoring the impact of diesel consumption in P&C is acceptable for the vast majority of cities.

**Table 3:** Comparison with data on total carbon emissions from city building operations based on the EBS splitting method.

Cities	Year of data	Carbon emissions from building operations (Mt CO_2_)		Difference between the two results
		This research	City EBS	
Tangshan	2016	17.23	18.26	−5.9%
Yangquan	2018	3.65	3.68	−0.8%
Shenyang	2018	38.98	41.54	−6.6%
Qingdao	2019	28.59	26.85	6.1%
Chenzhou	2017	3.48	3.63	−4.4%
Zhanjiang	2020	3.85	3.94	−2.6%
Hechi	2017	1.23	1.15	6.4%

Existing city-level carbon inventories and databases are mostly based on the production side of the equation, and it is not possible to disaggregate a complete consumption-based view of building carbon emissions. In this study, we conduct one-to-one comparisons of city data in three ways: First, we aggregated city data on total building carbon emissions to the provinces to compare with provincial data accounted for using the EBS methodology^42^. Second, we disaggregated direct carbon emissions from fossil energy for residential and service sectors from the 2020 city carbon emissions dataset compiled by the CCG to make a partial comparison. Thirdly, we accounted for the energy use of appliances in residential buildings based on the ownership of major energy-consuming household appliances and the electricity consumption of each appliance to compare with the end-use data from A&O.

When accounting for the validated data on energy use of household appliances, refrigerators, washing machines, televisions, and computers are the main sources of energy use among the traditional appliances essential for ordinary households, after excluding appliances used for space cooling, heating, and C&W. Therefore, these four appliances were selected, and the total electricity consumption per appliance was calculated based on their ownership and average annual electricity consumption for comparison with the electricity consumption figures for A&O derived from the split calculations in this study. The calculation formula is as follows:

22 $${E}_{elec}^{app{\prime} }=\sum _{n}{E}_{n}^{app{\prime} }=\sum _{n}\left({N}_{n}\times AE{C}_{n}\right)$$

23 $${N}_{n}=H\times AH{O}_{n}$$

where ${E}_{elec}^{app{\rm{{\prime} }}}$ is the annual electricity consumption of traditional home appliances in city residential buildings, *n* denotes four types of traditional home appliances, *N*_*n*_ is the ownership of home appliances, and *AEC*_*n*_ is the average annual electricity consumption of home appliances. The values are taken according to relevant surveys and studies, as shown in Table 4. *AHO*_*n*_ is the average household ownership of home appliances, and the values are taken from provincial data from the National Bureau of Statistics (NBS, https://data.stats.gov.cn/index.htm).

**Table 4:** Average annual electricity consumption of major traditional home appliances.

Household appliance	Average operating power (W)	Average daily usage hours (h)	Annual electricity consumption (kWh)	Note	Data sources
Washing machines	632.5	0.37	85.4		^27,29,66^
Refrigerators	150.0	19.92	419.5	The compressor running ratio is 1:1.6	
Televisions	200.0	3.33	243.1		
Computers	250.0	3.09	282.0		

**Fig. 3:**
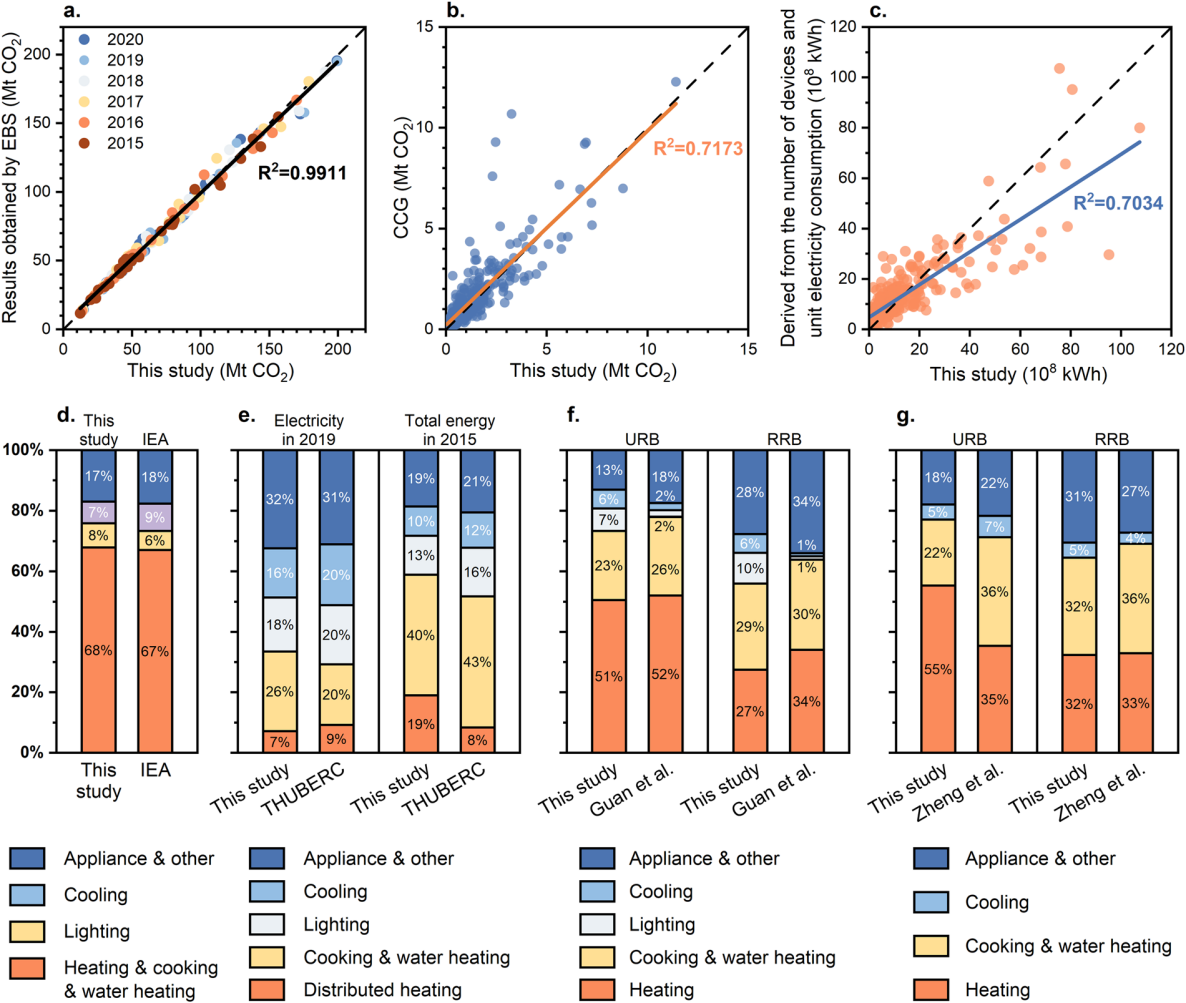
Comparison of operational energy consumption and carbon emissions in city buildings. (**a**) Comparison with provincial building carbon emissions accounted for using the EBS methodology^42^. Hainan and Qinghai provinces were not included in the comparison due to their missing city-level data. (**b**)Comparison with the China City Greenhouse Gas Working Group (CCG) for direct carbon emissions in city buildings in 2020. (**c**) Comparison of energy consumption for A&O electricity consumption in city residential buildings using different methods of accounting. (**d-g**) Comparison of summed end-use energy consumption and carbon emissions for city building operations. (**d**) Comparison with IEA data on total building energy consumption in China in 2020^63^. (**e**) Comparison with Tsinghua University Building Energy Research Centre (THUBERC) data on urban residential building energy consumption^26,27^. Energy consumption for central heating in the north is not included. (**f**) Comparison with the 2019 carbon emission data for residential buildings in China accounted for by Guan *et al*.^25^, where URB is urban residential buildings and RRB is rural residential buildings. (**g**) Comparison with the 2014 carbon emissions data for residential buildings in China accounted for by Zheng *et al*.^29^ Due to year constraints, the 2015 data from this study were used for comparison with the 2014 data from Zheng *et al*. For all data involving energy consumption in the comparison, electricity consumption was uniformly converted to standard coal using the heat equivalent method.

The comparison results are shown in Fig. 3a–c. Provincial-level comparisons reveal a significant alignment between the two datasets. Some variances primarily stem from the previously mentioned omission of diesel consumption and inconsistencies in the scope of accounting. For instance, we did not include data from counties directly under provincial jurisdiction. The consistency of direct emissions from fossil energy is also notably high, with variations predominantly attributable to coal consumption differences in Guizhou and Hunan. For A&O end-use, there is a partial deviation between the two sets of data due to the fact that only four traditional appliances are taken into account in the comparison methodology, while non-traditional appliances that are becoming more and more popular, such as dishwashers, projectors, smart toilet rings, and vacuums, have not been taken into account.

In terms of comparing data on building end-use energy consumption and carbon emissions, it is difficult to make comparisons for individual cities and provinces due to differences in the classification of building end-uses and energy categories in different studies. We therefore selected several studies based on large-scale research to compare with this study on a national scale after excluding the consumption of noncommodity energy such as biomass, converting electricity consumption consistently to standard coal using the heat equivalent method, and harmonizing the classification of building end uses. The comparison is shown in Fig. 3d–g. Overall, our accounting of the share of energy consumption and carbon emissions for each end use of the building at the summed national scale is closer to the results of other studies and surveys

### Correlation test

The building end-use data provided in this study is in the nature of filling in gaps in the current data and lacks complete city-level data for direct comparison. Therefore, we used city-level nighttime light data^56^, Heating Degree Day (HDD), and Cooling Degree Day (CDD) to perform correlation analyses of selected end-use energy.

**Fig. 4:**
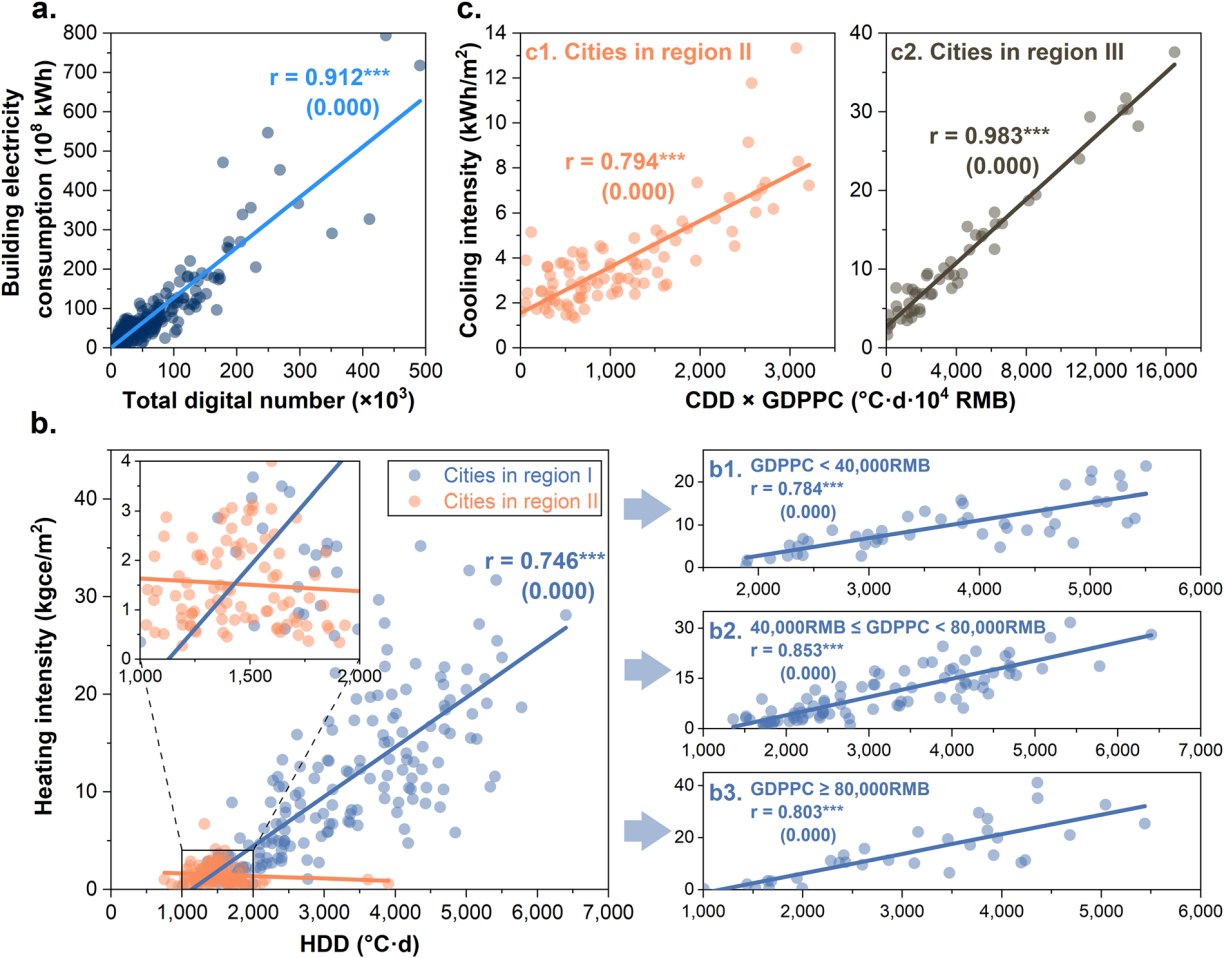
Results of correlation tests for electricity and selected end-use energy data for 2020. (**a**) Correlation between city nighttime light and building electricity consumption. (**b**) Correlation between city HDD and heating energy intensity (including central heating and distributed heating); furthermore, the cities in Region I are divided into three groups according to different economic levels (Gross Domestic Product per Capita (GDPPC)), which are GDPPC less than 40,000RMB (b1), GDPPC less than 80,000RMB (b2), and GDPPC greater than 80,000RMB (b3). (**c**) Correlation between the interaction term (consisting of the product of CDD and GDPPC) and the cooling energy intensity for cities in Region II (c1) and cities in Region III (c2). The p-values are in parentheses, and *** indicates significance at the 1% level.

The results of the analysis are shown in Fig. 4. The Pearson correlation coefficient (r) between the electricity consumption of city buildings and the total digital number (DN) value of lights at night is 0.912, which verifies the validity of the data on electricity consumption of city buildings. In Region I, the correlation coefficient between the energy intensity of city building heating and HDD is 0.746 with a significant p-value (0.000); in Region II, the energy intensity of distributed heating is more concentrated below 4 kgce/m^2^, and the correlation is not significant. Considering that residents’ heating activities are influenced by economic level, we controlled the variable of Gross Domestic Product per Capita (GDPPC) of cities using economic data provided by the *China City Statistical Yearbook*. The subgroup results show that the correlation coefficient for cities with GDPPC higher than 40,000RMB is higher than 0.8, and the partial correlation coefficient between building heating energy intensity and HDD rises to 0.800 for cities in Region I after controlling for the variable. For building cooling energy intensity, we use the product of CDD and GDPPC as the interaction term for correlation test, and the correlation coefficients are 0.794 and 0.983 in the cities in Region II and Region III, respectively, with significant p-values (0.000). Cities in Region I, which mostly have cooling energy intensities below 5 kWh/m^2^, are still significantly correlated with the interaction term (p-value = 0.006), but with a lower correlation coefficient (0.276).

### Uncertainty analysis

Uncertainty in greenhouse gas (GHG) emission inventories arises from conceptualization, modelling, data inputs and assumptions, where the uncertainty in conceptualization and modelling is difficult to quantify and resolve through technical means^57^. The carbon emissions from building operations in this study are all energy-related emissions, so we primarily consider uncertainty in activity data and emission factors to estimate uncertainty in the inventory.

The activity data in this study are mainly sourced from the statistical yearbooks of the National Bureau of Statistics and the statistical bulletins of various cities, which, although officially released by the government, remain highly uncertain and questionable due to the opaque nature of data collection, reporting, and validation methods in China’s statistical system^58,59^. In the current energy statistics system, it is common for the national total energy consumption to be inconsistent with the aggregated provincial energy consumption values, e.g., the national coal total consumption in 2020 was 4,049 million tonnes, while the aggregated provincial coal consumption was 4,640 million tonnes, a difference of 14.6%. The difference between the total energy consumption at the city level and the overall national figure is likely to be even greater^58^. We refer to the recommendations given in the IPCC Good Practice Guidance and set the uncertainty of activity data at 5% for LPG and NG^60^, for electricity consumption data, we set the uncertainty of data from officially published sources at 3%, and expanded the uncertainty of activity data derived from manual filling to 8%; and for coal, due to the lack of a well-produced statistical system, we set the uncertainty of its activity data at 10%.

The uncertainty of the emission factors was set at 3%, 1% and 2% for coal total, LPG and NG, respectively, with reference to the study by Shan *et al*.^10^ Electricity was determined to have an uncertainty of 3% as it is mainly generated by coal-fired power plants^61^.

**Fig. 5:**
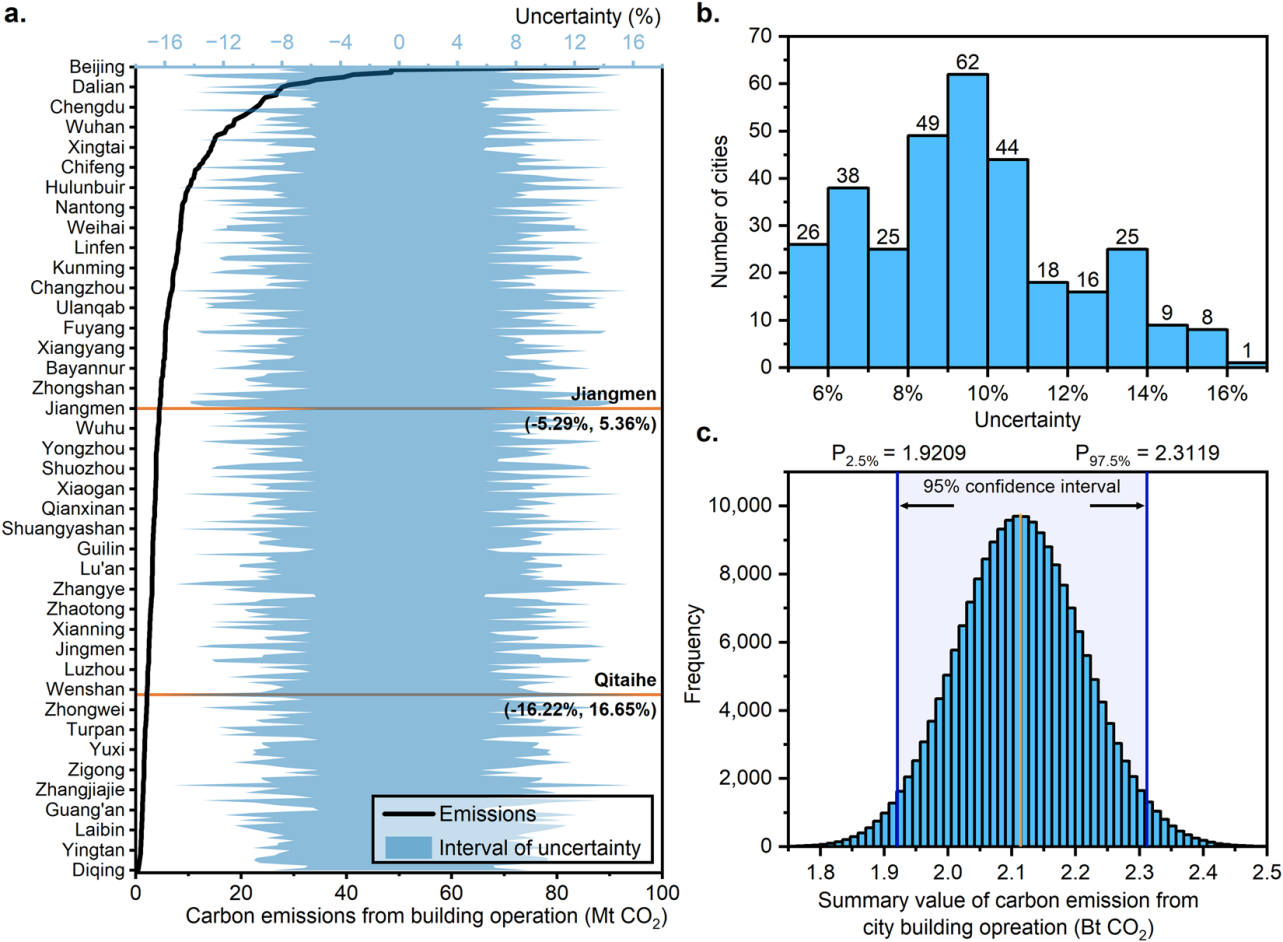
Uncertainty of operational carbon emissions from buildings at the city level in 2020. (**a**) Total building carbon emissions and uncertainty ranges for 321 cities. (**b**) Frequency distribution of uncertainty in city building emissions. (**c**) Monte Carlo analysis of total city building carbon emissions.

We used the IPCC-recommended Monte Carlo method to quantify the uncertainty in carbon emissions from city building operations^62^. We assumed that both input variables, activity data and emission factors, obeyed a normal distribution and obtained 200,000 estimates of building operational carbon emissions for each city using a random resampling 200,000 times in Python 3.10, using the parameters described above. The results of the Monte Carlo simulation for 2020 are shown in Fig. 5. The uncertainty range for total carbon emissions from city building operations in China in 2020 is −9.12% − 9.38% (95% confidence interval).

## Usage Notes

There are several uncertainties in our city-level building operations inventory of carbon emissions.

First, we have not explored the differences in fossil energy emission factors between provinces and cities in the selection of emission factors but have uniformly used the national recommended average emission factors and have used regional grid emission factors for electricity emission factors, which are not refined to the provincial level. In the future, if possible, we would like to apply the provincial and city-level emission factor data, updated with the year, to our emission inventory accounting.

Second, to address the issue of missing data on electricity consumption in some cities’ buildings and missing data on fossil energy consumption in P&C, we use interpolation and proportions methods for processing, which can lead to uncertainty in calculated city emissions. In the future, we will combine nighttime light data and high-resolution data from satellite observations to complete observations over longer time spans and obtain more accurate emission inventories.

Third, limited by the availability of data, we used provincial data on cooling intensity, proportion of electric water heaters, etc., for cities when accounting for building end-use energy consumption, which can mask differences between cities within a province. In the future, we will continue to combine temperature data and large-scale survey data to calibrate and adjust the building end-use energy consumption and carbon emissions data.

### Supplementary information

Supplementary Information

## Data Availability

All emission calculations involved in the method chapter are completed in Excel.
