# Supplementary material for: City-level building operation and end-use carbon emissions dataset from China for 2015–2020
Source: Sci Data. 2024 Jan 26;11:138. doi: 10.1038/s41597-024-02971-4 (PMC10817938; doi:10.1038/s41597-024-02971-4)
Supplement: Supplementary file 1 — Supplementary Information [file 41597_2024_2971_MOESM1_ESM.docx]

**City-level building operation and end-use carbon emissions dataset from China for 2015-2020**

Yanhui Yu^1^, Kairui You^1, 2^, Weiguang Cai^1^, Wei Feng^2^, Rui Li^1^, Qiqi Liu^1^, Liu Chen^1^, Yuan Liu^1^

**Affiliations**

1. School of Management Science and Real Estate, Chongqing University, Chongqing, 400044, China

2. Institute for Carbon Neutrality Technology, Chinese Academy of Sciences – Shenzhen Institute of Advanced Technology, Shenzhen, 518055, China

Corresponding author(s): Weiguang Cai ([wgcai@cqu.edu.cn](mailto:wgcai@cqu.edu.cn))

**Supplementary Information**

Supplementary Table S1 Cities with Special Zoning Attributes

| **Attribute** | **Province** | **City** |
| --- | --- | --- |
| Affiliated to northern provinces without centralized heating activities | Henan | Xinyang |
|  |  | Zhoukou |
|  | Shaanxi | Ankang |
| Affiliated to non-northern provinces with centralized heating activities | Anhui | Hefei |
|  |  | Huainan |
|  |  | Suzhou |
|  |  | Chuzhou |
|  | Hubei | Huangshi |
|  |  | Shiyan |
|  |  | Xiangyang |

Supplementary Table S2 Changes in city zoning

| **Province** | **City** | **Zoning change** |
| --- | --- | --- |
| Jiangsu | Xuzhou | Region I (2017-2020), Region II (2015-2016) |
|  | Nantong | Region I (2020), Region II (2015-2019) |
|  | Taizhou | Region I (2020), Region II (2015-2019) |
| Zhejiang | Hangzhou | Region I (2017), Region II (2015-2016, 2018-2020) |
|  | Ningbo | Region I (2015), Region II (2016-2020) |
|  | Jinhua | Region I (2015), Region II (2016-2020) |
| Anhui | Huaibei | Region I (2015-2016), Region II (2017-2020) |
|  | Anqing | Region I (2015-2016), Region II (2017-2020) |
|  | Fuyang | Region I (2015, 2017), Region II (2016, 2018-2020) |
|  | Chizhou | Region I (2015, 2017-2020), Region II (2016) |
| Fujian | Nanping | Region I (2017), Region III (2015-2016, 2018-2020) |
|  | Longyan | Region I (2017), Region III (2015-2016, 2018-2020) |
| Hubei | Jingmen | Region I (2019-2020), Region II (2015-2018) |
| Hunan | Xiangtan | Region I (2018), Region II (2015-2017, 2019-2020) |
|  | Yueyang | Region I (2015, 2017-2020), Region II (2016) |
|  | Yiyang | Region I (2019), Region II (2015-2018, 2020) |
| Sichuan | Chengdu | Region I (2017, 2019-2020), Region II (2015-2016, 2018) |
|  | Panzhihua | Region I (2018-2020), Region II (2015-2017) |
|  | Guangyuan | Region I (2018), Region II (2015-2017, 2019-2020) |
|  | Suining | Region I (2015, 2017), Region II (2016, 2018-2020) |
|  | Neijiang | Region I (2015, 2017), Region II (2016, 2018-2020) |
|  | Leshan | Region I (2020), Region II (2015-2019) |
|  | Liangshan | Region I (2015), Region II (2016-2020) |
| Guizhou | Liupanshui | Region I (2016-2020), Region II (2015) |
| Yunnan | Chuxiong | Region I (2017-2018), Region III (2015-2016, 2019-2020) |
|  | Diqing | Region I (2017-2020), Region II (2015-2016) |

Supplementary Table S3 Sources of month-by-month electricity consumption data at the provincial level in China

| **Province** | **Organization** | **Web Link** | **Note** |
| --- | --- | --- | --- |
| Beijing | National Bureau of Statistics | <https://data.stats.gov.cn/easyquery.htm?cn=E0101> | Use month-by-month generation for substitution. |
| Tianjin | National Bureau of Statistics | <https://data.stats.gov.cn/easyquery.htm?cn=E0101> | Use month-by-month generation for substitution. |
| Hebei | Shijiazhuang Municipal Bureau of Statistics | <http://tjj.sjz.gov.cn/col/1584345010372/index.html> | Use Shijiazhuang, the provincial capital city, instead. The data is reported separately each month and needs to be searched month by month. Keywords "Electricity consumption in the whole society". |
| Shanxi | The People's Government of shanxi Province | <http://www.shanxi.gov.cn/> | The data is reported separately each month and needs to be searched month by month. Keywords "Electricity consumption in the whole society". |
| Inner Mongolia | Statistics Bureau of Inner Mongolia Autonomous Region | <http://tj.nmg.gov.cn/tjyw/jpsj/> | Monthly statistical report |
| Liaoning | The People 's Government of Liaoning Province | <https://www.ln.gov.cn/web/index/index.shtml> | The data is reported separately each month and needs to be searched month by month. Keywords "Electricity consumption in the whole society". |
| Jilin | Statistical Bureau of Jilin | <http://tjj.jl.gov.cn/tjsj/> | The data is reported separately each month and needs to be searched month by month. Keywords "Electricity consumption in the whole society". |
| Heilongjiang | Heilongjiang Bureau of Statistics | <http://tjj.hlj.gov.cn/tjj/c106783/common_zfxxgk.shtml> |  |
| Shanghai | Shanghai Municipal Commission of Economy and Informatization | <https://jjyx.sheitc.sh.gov.cn/list.jsp?channelId=02> |  |
| Jiangsu | Statistical Bureau of Jiangsu | <http://tj.jiangsu.gov.cn/col/col85905/index.html> |  |
| Zhejiang | Statistical Bureau of Zhejiang | <https://mapi.zjzwfw.gov.cn/web/mgop/gov-open/zj/2001941911/reserved/index.html#/publishingIndicatorSearch/jdzb?themeCode=THEME_Y_33&folderId=THEME_Y_33urn%3Addi%3AZJJCKSTAT%3Ada1a4200-df1b-4c8e-8178-2c8aafa1741b%3A1> |  |
| Anhui | Statistical Bureau of Anhui | <http://tjj.ah.gov.cn/ssah/qwfbjd/jdsj/index.html> |  |
| Fujian | - | - | Chinese web search. Keywords "Year-Month-Province-Electricity consumption in the whole society". |
| Jiangxi | Jiangxi Provincial Bureau of Statistics | <http://tjj.jiangxi.gov.cn/> | The data is reported separately each month and needs to be searched month by month. Keywords "Electricity consumption in the whole society". |
| Shandong | Jinan Municipal Bureau of Statistics | <http://jntj.jinan.gov.cn/col/col18253/index.html?uid=32212&pageNum=1> | Use Jinan, the provincial capital city, instead. The data is reported separately each month and needs to be searched month by month. Keywords "Electricity consumption in the whole society". |
| Henan | Henan Energy Regulatory Office of National Energy Administration of the People's Republic of China | <http://henb.nea.gov.cn/frontIndex/contentlistByColumnIddd.do?columnId=Col_jgdt&contentPage.pageSize=20> |  |
| Hubei | Hubei Provincial Statistics Bureau | <http://tjj.hubei.gov.cn/tjsj/sjkscx/tjyb/wnyb/tjyb2020/> |  |
| Hunan | People's Government of Hunan Province | <http://www.hunan.gov.cn/hnszf/zfsj/zfsj.html> | The data is reported separately each month and needs to be searched month by month. Keywords "Electricity consumption in the whole society". |
| Guangdong | Statistical Bureau of Guangdong | <http://stats.gd.gov.cn/gmjjzyzb/index_2.html> |  |
| Guangxi | Statistical Bureau of Guangxi | <http://tjj.gxzf.gov.cn/tjsj/jdsj/qqydsj/2020_qqydjs/index.shtml> |  |
| Hainan | Hainan Provincial Bureau of Statistics | <https://stats.hainan.gov.cn/tjj/tjsu/jdsj/> |  |
| Chongqing | Chongqing Economic and Information Technology Commission | <https://jjxxw.cq.gov.cn/> | The data is reported separately each month and needs to be searched month by month. Keywords "Electricity consumption". |
| Sichuan | Chengdu Municipal Statistics Bureau | <https://cdstats.chengdu.gov.cn/cdstjj/c155009/list.shtml> | Use Chengdu, the provincial capital city, instead. Chengdu Statistical Yearbook: 8-11 Monthly Total Electricity Consumption |
| Guizhou | Guizhou Energy Administration | <http://nyj.guizhou.gov.cn/zwgk/xxgkml/zdlyxx/tjsj/index.html> | The data is reported separately each month and needs to be searched month by month. Keywords "Electricity consumption in the whole society". |
| Yunnan | Energy Administration of Yunnan Province | <http://nyj.yn.gov.cn/nydt/ynnydt/> | The data is reported separately each month and needs to be searched month by month. Keywords "Electricity consumption in the whole society". |
| Shaanxi | Statistical Bureau of Shaanxi | <http://tjj.shaanxi.gov.cn/> | The data is reported separately each month and needs to be searched month by month. Keywords "Energy consumption". |
| Gansu | Department of Industry and Information Technology of Gansu Province | <http://gxt.gansu.gov.cn/gxt/jjyx/infolist_nonav.shtml> |  |
| Qinghai | Qinghai Provincial Department of Industry and Information Technology | <http://gxgz.qinghai.gov.cn/html/59/List.html> |  |
| Ningxia | Statistics Bureau of Ningxia Hui Autonomous Region | <https://tj.nx.gov.cn/> | The data is reported separately each month and needs to be searched month by month. Keywords "Energy consumption". |
| Xinjiang | Statistic Bureau of Xinjiang Uygur Autonomous Region | <http://tjj.xinjiang.gov.cn/> | The data is reported separately each month and needs to be searched month by month. Keywords "Energy production and consumption". |
